# ZnTe Crystal Multimode Cryogenic Thermometry Using Raman and Luminescence Spectroscopy

**DOI:** 10.3390/ma16031311

**Published:** 2023-02-03

**Authors:** Evgenii V. Borisov, Alexey A. Kalinichev, Ilya E. Kolesnikov

**Affiliations:** Center for Optical and Laser Materials Research, St. Petersburg University, Universitetskaya nab. 7-9, 199034 St. Petersburg, Russia

**Keywords:** ZnTe, optical thermometry, photoluminescence, Raman spectrum, cryogenic temperature, multimode sensing

## Abstract

In this study, ZnTe crystal was applied to provide precise thermal sensing for cryogenic temperatures. Multiple techniques, namely Raman and photoluminescence spectroscopies, were used to broaden the operating temperature range and improve the reliability of the proposed thermometers. Raman-based temperature sensing could be applied in the range of 20–100 K, while luminescence-based thermometry could be utilized in a narrower range of 20–70 K. However, the latter strategy provides better relative thermal sensitivity and temperature resolution. The best thermal performances based on a single temperature-dependent parameter attain *S_r_* = 3.82% K^−1^ and Δ*T* = 0.12 K at *T* = 50 K. The synergy between multiple linear regression and multiparametric thermal sensing demonstrated for Raman-based thermometry results in a ten-fold improvement of _Sr_ and a two-fold enhancement of Δ*T*. All studies performed testify that the ZnTe crystal is a promising multimode contactless optical sensor for cryogenic thermometry.

## 1. Introduction

Accurate temperature measurements are crucial for the majority of human activities, including science, medicine, agriculture, industry, and aerospace [1,2,3,4,5]. Traditional thermometers, such as liquid-filled and bimetallic thermometers, thermocouples, and thermo-resistance, generally require physical contact and thermal transmission, severely restricting their applications in moving objects, hazardous and inaccessible locations, or micro/nanoscale [6,7,8]. To overcome these limitations, remote temperature sensing based on monitoring the changes in the optical properties of samples was proposed [9,10,11,12,13]. Optical thermometry can be realized via the following methods: optical interferometry, near-field optical scanning microscopy, Raman scattering, and luminescence spectroscopy [7,14,15].

Optical interferometry could be used for high-resolution thermal sensing. The principle of optical interferometry is as follows: it monitors changes in the refractive index of a fluid triggered by temperature. The technique utilizes an even illumination source (Köhler illumination) and a wavefront analyzer to provide temperature mapping [9,16]. Baffou et al. demonstrated optical interferometry for temperature sensing by measuring the heat dissipated by a gold microwire, as well as bubble formation from plasmonic nanostructures in water [17,18]. Limitations of optical thermometry are the study of samples immersed in fluids and the need for a model to define the refractive index of the fluid [9].

Raman and luminescence thermometers provide contactless temperature sensing with suitable spatial resolution and 3D mapping. Raman spectroscopy can be applied to a wide range of samples and experimental conditions, but it requires a long integration time to record the optical signal, which is usually weak. The main advantages of this method are the rather simple sample preparation, the sufficiency of a small amount of material, and the applicability in almost all environments, even during chemical reactions or under extreme pressure and temperature conditions. Raman spectroscopy works over a wide temperature range but is limited at high temperatures due to black-body radiation (above ~1000 K). In principle, each material is potentially a thermal sensor in terms of Raman light scattering, and therefore, this method can be applied in many different situations. However, limitations arise because of the need for sample transparency and the weak intensity of Raman signals. Luminescence thermometry attracts more attention because it combines high relative thermal sensitivity and spatial resolution with short acquisition times [19,20,21]. Temperature-induced changes in photoluminescence parameters that include intensity (or intensity ratio of two bands), lifetime, bandwidth, spectral position, and polarization can be used for luminescence thermal sensing [22,23,24,25,26,27]. The vast majority of known luminescence thermometers utilize a single parameter to provide temperature sensing. However, it has been reported that the use of a combination of distinct thermometric parameters could improve the reliability of thermometers [28,29,30,31,32]. In addition to reliability, this multiparametric approach could significantly improve the relative thermal sensitivity and temperature resolution of the sensor using a multiple linear regression-based strategy proposed by Carlos et al. [33,34]. Another strategy to use several temperature-dependent luminescence parameters for the enhancement of thermometric characteristics has been reported by Khodasevich et al. [35]. Authors developed a multivariate model of temperature calibration by the spectra of green upconversion fluorescence of GeO_2_–Na_2_O–Yb_2_O_3_–MgO–La_2_O_3_–Er_2_O_3_ glass ceramics based on the principal component analysis, cluster analysis, and the interval projection to latent structures.

Herein, we studied ZnTe crystal as a multimode optical thermal sensor in the cryogenic temperature range. Raman and photoluminescence spectroscopies have been successfully applied for thermometry using various temperature-dependent parameters, including the ratio between Raman modes, the luminescence intensity ratio, the spectral position, and the bandwidth of the emission line. The advantage of multiparametric thermometry was realized through the application of multiple linear regression, which led to significant enhancement of thermometric performances.

## 2. Experimental

The sample under study was a thick layer of zinc telluride film 3 × 3 mm in size and approximately 2 mm in height, grown by the MOCVD technique on a GaAs substrate. Dimethylzinc (DMZn) and diethyltellurium (DETe) were used as precursors. The ZnTe film was grown in a cubic phase with a zinc blend structure (sphalerite). The lattice constant a = 0.61 nm [36,37]. The crystalline cell is face-centered, and the primitive cell contains two atoms.

Raman spectroscopy was performed using a T64000 research-grade spectrometer (Horiba Scientific, Kyoto, Japan) with a 532 nm solid-state laser as an excitation source and in a backscattering geometry (scattering angle of 180°). Signal detection was performed via Peltier-cooled CCD matrix Synapse (Horiba Scientific, Kyoto, Japan). Measurements were carried out in a single spectrometer mode with 1800 gr/mm diffraction grating. Laser radiation was focused onto the sample surface using a 50× microobjective (NA 0.6). Raman signals of the 1LO, 2LO, and 3LO modes were recorded using different acquisition times (namely 2, 10, and 20 s) due to a significant difference in their intensities. To improve the signal-to-noise ratio, 8 repetitions were carried out for each measurement. The data presented take into account different acquisition times to provide a fair calculation of the intensity ratios.

Emission spectra were obtained using the same spectrometer with a 514 nm diode laser as an excitation source (power density 350 kW/cm^2^). Laser radiation was focused onto the sample surface using a 50× microobjective (NA 0.6). The chosen power density was weak enough not to disturb the sample temperature during irradiation. The typical acquisition time for emission measurements varies from 1 to 3 s, depending on the sample temperature. To improve the signal-to-noise ratio, 4 repetitions were carried out for each measurement. All other experimental parameters used were the same as in Raman measurements. The data provided were normalized to an acquisition time. The sample was placed in a helium cryostat (CryoIndustries, Manchester, NH, USA) to carry out temperature measurements. The operating temperature regime was 20–100 K, and the temperature stability was 0.05 K. During thermal studies, a 5 min gap was used to stabilize the temperature of the sample prior to each measurement.

## 3. Results and Discussion

**Figure 1:**
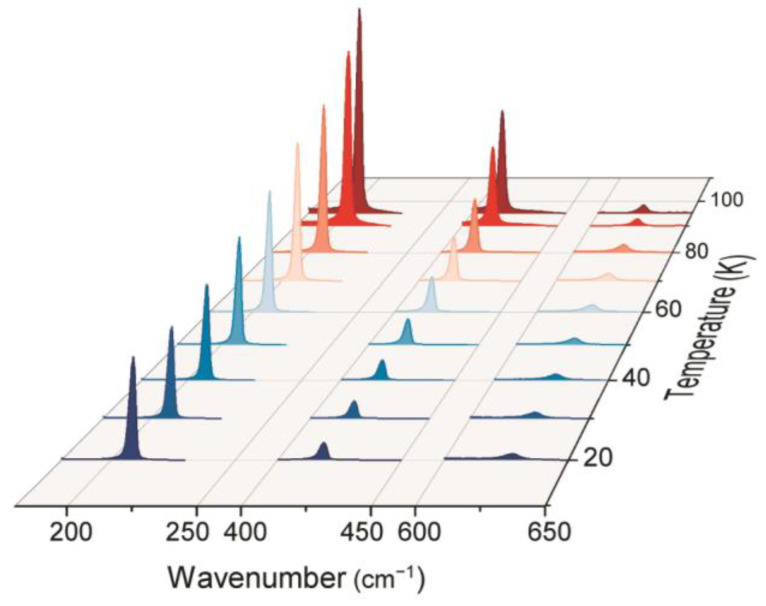
Raman spectra of the ZnTe film measured in the temperature range of 20–100 K.

Raman spectra of the ZnTe sample measured within the spectral range of 180–650 cm^−1^ at different temperatures (20–100 K) are shown in Figure 1. In accordance with the group theory, in the common case, six phonon modes occur in ZnTe sphalerite crystal, three of them are acoustic, and three are optical, three-fold degenerate. Thus, one should observe only one optical phonon mode in the Raman spectrum as well as its overtones. Furthermore, crystalline tellurium, which can aggregate in the grown ZnTe layers, contributes to vibrational spectra in the low-frequency range (below 150 cm^−1^) [38,39]. The observed ZnTe vibrational lines centered at 208.9, 418.8, and 627.9 cm^−1^ (at *T* = 20 K) are assigned to 1LO, 2LO, and 3LO modes, respectively. No crystalline tellurium modes were observed in the studied spectral region. It can be seen that temperature increase leads to the broadening and red shift of all Raman bands. In addition, temperature growth results in an increase in Raman intensity.

**Figure 2:**
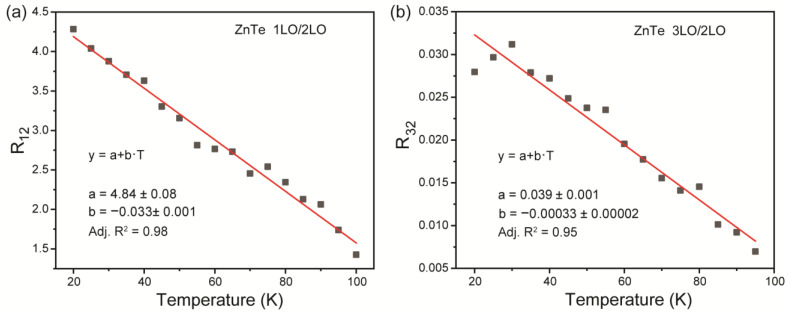
Temperature evolution of (**a**) R_12_ and (**b**) R_32_.

Careful analysis of thermally induced change of ZnTe Raman spectra shows that ratiometric technique between different observed vibrational lines can provide contactless temperature sensing. For example, the intensity ratio between 1LO and 2LO intensities (R_12_) can be utilized as a temperature-dependent parameter for optical thermometry. The experimental values of R_12_ as a function of temperature are presented in Figure 2a. The observed R_12_ temperature dependence can be accurately fitted by a pseudo-linear function with suitable quality Adj. R^2^ = 0.98, which facilitates defining temperature from the calculated ratio. Another temperature-sensitive parameter proposed is the intensity ratio between 3LO and 2LO lines—R_32_. Similar to the former case, R_32_ experimental values were approximated with a linear function with slightly worse quality. Both ratiometric approaches prove the possibility of using Raman spectra of ZnTe for optical thermal sensing. However, R_12_ provides thermometry within the temperature range of 20–100 K, whereas R_32_ allows thermal sensing in the region of 20–95 K. This fact could be explained by an indistinguishable intensity of the 3LO mode at 100 K.

**Figure 3:**
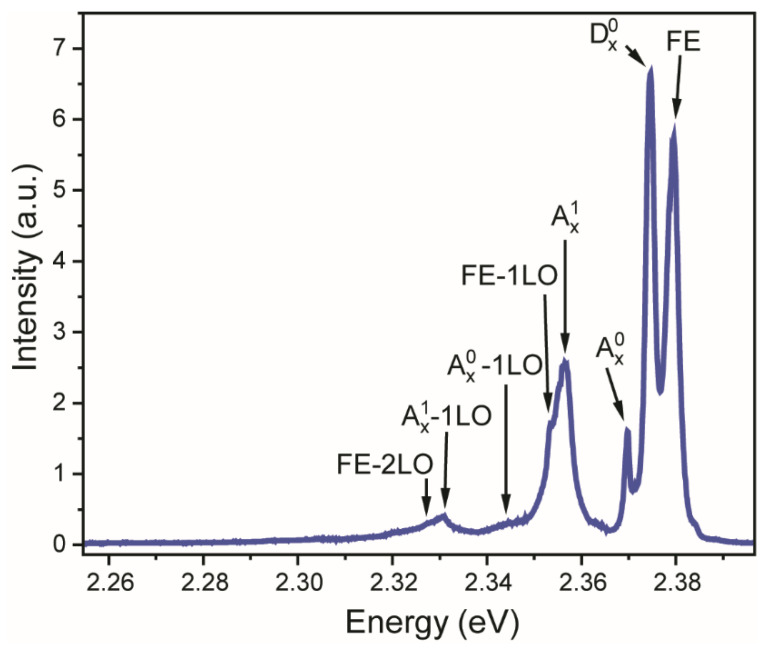
Emission spectrum of ZnTe film under 514 nm excitation at *T* = 20 K.

In addition to Raman spectroscopy, luminescence measurements are widely known as one of the most perspective methods to provide contactless optical thermometry. Zinc telluride is a direct band semiconductor with a 2.26 eV band gap [40,41]. The valence band consists of two sub-bands with different effective masses; however, the maxima corresponding to the light-hole and heavy-hole excitons cannot be observed in low-temperature photoluminescence spectra of bulk and thick ZnTe films. Figure 3 displays ZnTe emission spectra upon 514 nm excitation measured at 20 K. We focused on ZnTe exciton luminescence bands as these lines undergo the largest thermally induced change. All observed emission bands are ascribed to the corresponding transitions (Table 1).

**Figure 4:**
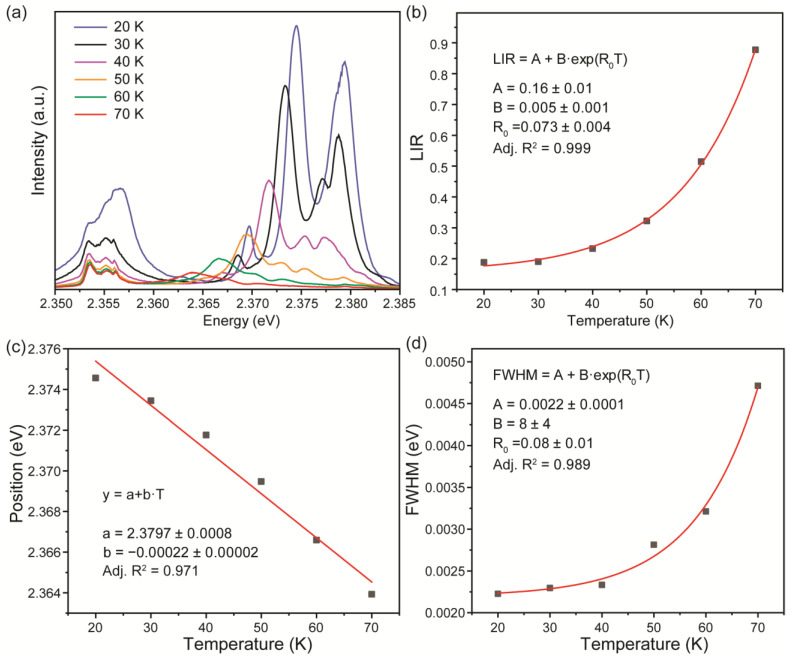
(**a**) Magnified area of ZnTe film emission spectrum; (**b**) LIR temperature dependence; temperature evolution of (**c**) $D_{X}^{0}$ spectral line position and (**d**) bandwidth of $D_{X}^{0}$ spectral line.

Further, emission spectra of the ZnTe sample were measured at different temperatures (Figure 4a). One can see significant thermal quenching of both FE-1LO and $D_{X}^{0}$ bands accompanied by a red shift of the latter one, along with a temperature increase. The spectroscopic parameters of emission bands, including intensity and line position, were obtained from the deconvolution procedure, which was performed for each temperature. The best fit was obtained by using the Voight line shape for both bands. The luminescence intensity ratio between the FE-1LO and $D_{X}^{0}$ transitions was proposed as a temperature-dependent parameter for optical thermometry. As can be seen from Figure 4b, LIR could provide temperature sensing within the range of 20–70 K. The experimental data were successfully fitted with an exponential function: $LIR=\frac{I_{FE-1LO}}{I_{D_{X}^{0}}}=A+B\cdot e^{R_{0}T}$, where *B*, *C*, and *R*_0_ are temperature-independent constants. The approximation used should be considered a phenomenological function.

In addition to LIR, the spectral line position and bandwidth of the $D_{X}^{0}$ band could be sensitive parameters in the same temperature range (Figure 4c,d). One can see that the spectral line displays monotonic behavior with temperature, which results in a gradual red shift along with temperature growth. Temperature increase led to the broadening of luminescence bands, and the bandwidth of the $D_{X}^{0}$ line was approximated using the same exponential function as LIR.

To date, several models have been developed that describe the temperature dependence of the luminescence bandwidth for transition metals and lanthanides [48]. $D_{X}^{0}$ line monitored here is assigned to exciton band and could have significantly different temperature behavior. Rudin et al. reported theoretical model for temperature behavior of exciton band in semiconductor, but it is too complex to be used for practical application [49]. Therefore, the obtained experimental data were fitted with a simple phenomenological exponential equation.

**Figure 5:**
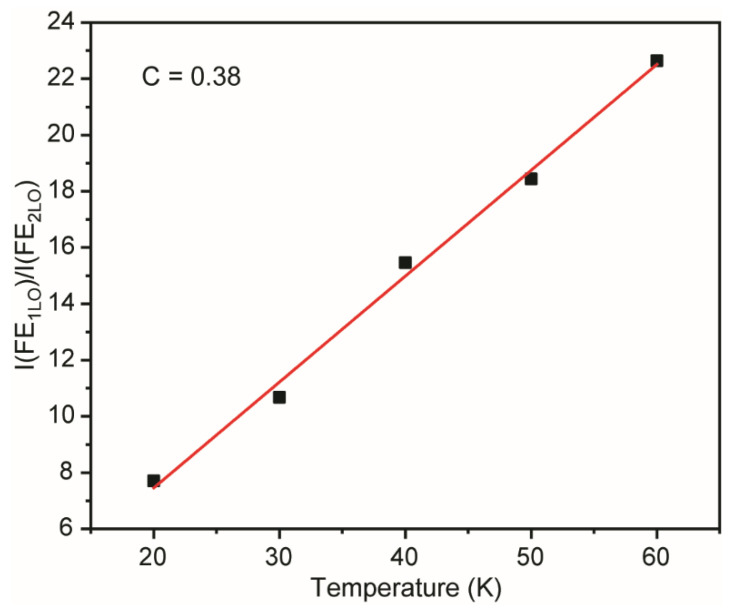
First and second phonon replica intensity linear approximation.

According to the observed data, the temperature dependence of free exciton first and second phonon replica intensity ratio linearly increases within the range 20–60 K (Figure 5). Similar data were acquired for gallium nitride, cadmium sulfide, and cadmium selenide [50,51]. In the case of free exciton radiative annihilation, only phonons with the wave vector close to the zero point participate in an interaction with excitons. However, considering the second phonon replica, the sum of two phonons wave vectors must be close to zero in order to interact with excitons. Along with the temperature increase, a bigger number of phonons in the first replica participate in the radiative annihilation, while the number of phonons corresponding to the second replica remains unchanged. Therefore, an equation could be derived $\frac{I(1LO)}{I(2LO)}=CT$, where *T* is temperature, and *C* is a coefficient, characterizing the grown layers quality. The obtained value *C* = 0.38 makes one conclude that synthesized ZnTe samples have a rather suitable quality [51].

**Figure 6:**
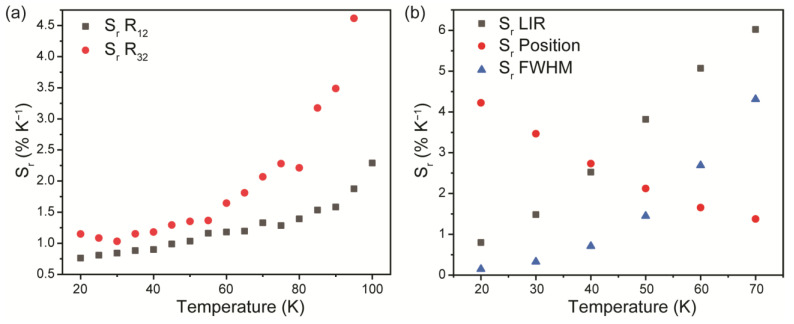
Temperature dependence of *S_r_* values for (**a**) Raman and (**b**) luminescence-based optical thermometry.

Thermometric performances of the studied ZnTe thermometer were assessed in terms of relative thermal sensitivity and temperature resolution. Relative thermal sensitivity, *S_r_*, was introduced to provide a fair comparison between various types of thermometers irrespective of their nature (e.g., mechanical, electrical, optical). *S_r_* could be obtained using the following equation: $S_{r}=\frac{1}{\Lambda }\left|\frac{\partial \Lambda }{\partial T}\right|$, where *Λ* is the monitored temperature-dependent parameter. Thermal sensitivity depends on temperature, so Figure 6 shows *S_r_* values calculated for different proposed sensing parameters as a function of temperature. It can be seen that relative thermal sensitivity increases with temperature growth, except for thermometry, based on the position of the spectral line. The best *S_r_* value of 6.0% K^−1^@70 K was achieved using LIR as a temperature-dependent parameter.

The temperature resolution, Δ*T*, which defines the accuracy of the thermometer, can be found via several methods [52]. Temperature resolution for ratiometric approaches was calculated from the expression: $∆T=\frac{1}{S_{r}}\frac{\delta R}{R}$, where δR is the uncertainty in the R value that may be defined as $\delta R=\delta \left(\frac{I_{1}}{I_{2}}\right)=\sqrt{{\left(\frac{\delta I_{1}}{I_{2}}\right)}^{2}+{\left(\frac{I_{1}\delta I_{2}}{{I_{2}}^{2}}\right)}^{2}}=\frac{I_{1}}{I_{2}}\sqrt{{\left(\frac{\delta I_{1}}{I_{1}}\right)}^{2}+{\left(\frac{\delta I_{2}}{I_{2}}\right)}^{2}}$ [53,54]. Finally, $∆T=\frac{1}{S_{r}}\sqrt{{\left(\frac{\delta I_{1}}{I_{1}}\right)}^{2}+{\left(\frac{\delta I_{2}}{I_{2}}\right)}^{2}}$, where the $\delta I_{1}$ and $\delta I_{2}$ are obtained from the integrated intensity of the noise.

Recently, Carlos et al. clearly demonstrated that multiparametric thermal sensing could improve not only the reliability of luminescence thermometers but also significantly enhance the relative thermal sensitivity and temperature resolution by using multiple linear regression (MLR) [33]. The main idea is as follows: if a thermometer has several thermometric parameters that vary linearly with temperature, i.e., Δ_1_, Δ_2_, …, Δ*_n_*, then the temperature can be expressed as a function of each Δ, i.e., *T* = *f*(Δ_1_, Δ_2_, …, Δ*_n_*): $T=\beta_{0}+\sum_{i=1}^{n}\beta_{i}{∆}_{i}+\varepsilon$, where $\beta_{0}$ is the intercept, $\beta_{i}$ (*i* = 1, …, *n*) is the slope of each thermometric parameter Δ*_i_* (explanatory variable *i*), and ε is the residual [33,55]. Thus, thermometric performances could be rewritten as follows: $S_{r}=\sqrt{\sum_{i=1}^{n}{\left(\frac{1}{{∆}_{i}}\left|\frac{\partial {∆}_{i}}{\partial T}\right|\right)}^{2}}=\sqrt{\sum_{i=1}^{n}{\left({∆}_{i}\left|\frac{\partial T}{\partial {∆}_{i}}\right|\right)}^{-2}}=\sqrt{\sum_{i=1}^{n}{\left({∆}_{i}\beta_{i}\right)}^{-2}}$ and $∆T=\frac{1}{S_{r}}\sqrt{\sum_{i=1}^{n}{\left(\frac{\delta {∆}_{i}}{{∆}_{i}}\right)}^{2}}$, where *δ*Δ*_i_*/Δ*_i_* is the relative uncertainty in each thermometric parameter.

As a proof of concept, the MLR approach was applied to experimental data obtained from Raman measurements with two distinct Δ*_i_* parameters previously defined as R_12_ and R_32_. Table 2 lists relative thermal sensitivity and temperature resolution based on different thermometry strategies, which were obtained using standard and MLR approaches. The synergy between MLR and multiparametric thermal sensing results in about a ten-fold improvement of *S_r_* and a two-fold improvement of Δ*T*. Noteworthy, all suggested thermometry approaches provide sub-degree temperature sensing at 50 K. The best temperature resolution attained with MLR exceeds 0.1 K.

## 4. Conclusions

In summary, we successfully demonstrate the ZnTe sample as a multimode optical thermal sensor for cryogenic temperatures. Contactless thermometry was performed using Raman and luminescence spectroscopy. Two different intensity ratios between optical vibration modes were utilized for sensing in a range of 20–100 K. Luminescence thermometry also provides a multiparametric approach using LIR between FE-1LO and $D_{X}^{0}$ transitions, spectral line position, and bandwidth of $D_{X}^{0}$ band. The thermometric performances of the ZnTe sample were assessed via relative thermal sensitivity and temperature resolution, showing suitable prospects for use in real applications. The best sensitivity based on a single temperature-dependent parameter attains 3.82% K^−1^@50 K, while temperature resolution was found to be sub-degree for all proposed parameters. The synergy between multiple linear regression and multiparametric thermal sensing allows the thermometric performances of ZnTe temperature sensors to be significantly enhanced. Ten-fold improvement of *S_r_* and two-fold enhancement of Δ*T* make the thermometric performances unprecedentedly high for contactless thermometers using Raman spectroscopy.

## Figures and Tables

**Table 1:** Assignment of ZnTe exciton luminescence bands, *T* = 20 K [42,43,44,45,46,47].

Emission Assignment	Symbol	Energy Position, eV
Free exciton	FE	2.3796
Exciton bound to neutral donor	$D_{X}^{0}$	2.3746
Exciton bound to neutral acceptor	$A_{X}^{0}$	2.3698
Exciton bound to charged acceptor	$A_{X}^{1}$	2.3567
First phonon replica of free exciton	FE-1LO	2.3535
First phonon replica of exciton bound to neutral acceptor	$A_{X}^{0}$-1LO	2.3439
First phonon replica of exciton bound to charged acceptor	$A_{X}^{1}$-1LO	2.3307
Second phonon replica of free exciton	FE-1LO	2.3273

**Table 2:** Thermometric performances of ZnTe crystal using different sensing parameters (*T* = 50 K).

Material	Sensing Parameter	*S_r_* (% K)	Δ*T* (K)
ZnTe	R_12_ (Raman)	1.04	0.16
	R_32_ (Raman)	1.35	0.79
	LIR (luminescence)	3.82	0.12
	Position (luminescence)	2.12	–
	FWHM (luminescence)	1.45	–
	MLR (Raman)	15.44	0.07

## Data Availability

Not applicable.
